# Relationship Between Serum Cystatin C and Creatinine or Dialysis Adequacy in Patients on Chronic Maintenance Hemodialysis

**DOI:** 10.5812/numonthly.4934

**Published:** 2013-03-30

**Authors:** Zhinoos Khorgami, Alireza Abdollahi, Samaneh Soleimani, Farrokhlagha Ahamadi, Mitra Mahdavi-Mazdeh

**Affiliations:** 1 Iranian Tissue Bank Research and Preparation Center, Tehran University of Medical Sciences, Tehran, IR Iran; 2 Department of Pathology, Imam Khomeini Hospital, Tehran University of Medical Sciences, Tehran, IR Iran; 3 Nephrology Research Center, Tehran University of Medical Sciences, Tehran, IR Iran

**Keywords:** Cystatin C, Glomerular Filtration Rate, Creatinine, Renal Dialysis

## Abstract

**Background:**

Glomerular filtration rate (GFR) is widely estimated by serum creatinine based equations such as Cockcroft-Gault (CG) standardized for body surface, and an abbreviated formula derived from MDRD (modification of diet in renal disease) study. However, some studies suggested that creatinine based estimation of GFR formula can be replaced by cystatin C based formula.

**Objectives:**

The aim of this study was to determine whether cystatin C based equation could be used as an indicator for renal function in hemodialysis patients compared to MDRD equation; and whether cystatin C, a dialyzable molecule, was related to Kt/V, the marker for dialysis adequacy.

**Patients and Methods:**

In this cross-sectional study, 98 patients on chronic hemodialysis were included. Plasma levels of urea and creatinine were measured before and after dialysis, and cystatin C was measured before dialysis. GFR was calculated and compared.

**Results:**

The mean age of patients was 55.50 ± 16.10 (24-86) years and 66 cases were male (67.3%). The GFR was estimated at 6.05 ± 2.36 and 5.83 ± 2.19 cc/min by MDRD and cystatin C based formulas, respectively, with a significant correlation (r = 0.51; P < 0.001). Serum cystatin C level was 9.74 ± 2.47 mg/L which showed significant reverse correlation with both MDRD (r = -0.46; P < 0.001) and cystatin C based formulas (r = -0.87; P < 0.001). Neither creatinine nor serum cystatin C showed correlation with Kt/V, as the marker of dialysis adequacy.

**Conclusions:**

Serum cystatin C may be considered as an indicator of renal function in patients under maintenance hemodialysis.

## 1. Background

Glomerular filtration rate (GFR), one of the most important parameters for assessment of renal function, is widely estimated by serum creatinine based equations of Cockcroft-Gault (CG) standardized for body surface, and modification of diet in renal disease (MDRD) formula. Both equations take parameters of serum creatinine, age, and gender into account. As creatinine production is affected by age, muscle mass, gender, medications, and catabolic state, the serum cystatin C based equations were proposed for GFR estimation (1, 2), especially because it has been recently shown that ethnicity coefficients did not seem to be necessary (3).

Cystatin C, a medium size molecule (13 k Daltons), is a cysteine proteinase inhibitor. It is synthesized by all nucleated cells with constant production rate, freely filterable through the glomerulus due to its small size, and metabolized by proximal tubular cells (2, 4). It should be mentioned that there are studies showing the relation between serum cystatin C and gender or age (5, 6).

Randers et al. studied serum cystatin C, serum and urine creatinine, and GFR by 99mTc-DTPA clearance technique in 76 patients with various kidney diseases and serum creatinine less than 500 mmol/L, and 61 dialysis patients (40 on hemodialysis and 21 on peritoneal dialysis) in 1998. Similar to all plasma clearance techniques, 99mTc-DTPA clearance technique is reliable in GFR values higher than 20-30 mL/min. They found its significant linear relationship with 1/cystatin C and 1/creatinine in those with GFR higher than 30 mL/min (7).

Later, many other studies showed that serum cystatin C is a better estimator of GFR than serum creatinine in different populations (5, 6, 8, 9). As mentioned before, most of the studies focused on patients with mild to moderate renal dysfunction, and the various developed cystatin C based equations have still questionable accuracy compared mainly to radionuclide estimation of GFR (10). Andersen et al. reviewed clinical studies in children evaluating serum cystatin C, cystatin C based formulas, and plasma creatinine or creatinine based formulas against an exogenous reference method and concluded that cystatin C based formulas are comparable with creatinine based formulas (11).

Furthermore, small size of cystatin C may make it dialyzable and possibly a marker for middle molecular size toxin removal, as Al-Malki et al. postulated. They showed that the mean level of serum cystatin C in those on chronic dialysis was influenced not only by method but also by intensity of dialysis. They mentioned that it may be able to have some role in monitoring the adequacy of dialysis (12).

## 2. Objectives

The aim of this study was to determine whether cystatin C based equation could be used as an indicator for renal function in hemodialysis patients compared to MDRD equation, and whether cystatin C, a dialyzable molecule, was related to Kt/V, a marker of dialysis adequacy.

## 3. Patients and Methods

In this cross-sectional study, 98 patients on maintenance hemodialysis in dialysis ward of Imam Khomeini hospital, Tehran, Iran, during April 2010 to June 2010 were enrolled. All patients were stable at the time of study and on regular thrice-weekly 4-hour sessions hemodialysis with synthetic polysulfone dialyzers for at least 4 months. Venous blood samples were collected from each patient, before and after dialysis. The collected samples before the dialysis were used to measure urea, creatinine, cystatin C, and albumin. The plasma urea samples taken after the dialysis were used to measure single pool (sp) Kt/V. Blood samples were centrifuged at 1500 × g for 10 min at 4 ºC. Creatinine was measured by enzymatic-colorimetric kinetic using Elitech diagnostic kit from ELITech Group France. Urea was measured by Enzymatic-UV kinetic using Elitech diagnostic kit from ELITech Group France. Cystatin C was measured by immunoassay method using Gentian kit Gentian Services LTD. The mean values of creatinine for each patient were used in MDRD formula (eGFR = 186 × Serum Creatinine (mg/Dl) ^-1.154^ × Age (years) ^-0.203^ × [1.212 if Black ] × [0.742 if Female]) and eGFR cysC was calculated by chronic kidney disease epidemiology collaboration (CKD-EPI) equation: eGFR = 127.7 (cystatin C in mg/L) ^- 1.179^ (age) ^- 0.139^ (0.91 if female).

All statistical analyses were conducted with SPSS, version 19. Baseline characteristics were presented as the mean ± SD. The Spearman correlation coefficient was used to study correlations between variables. P < 0.05 was regarded as statistically significant.

## 4. Results

The mean age of patients was 55.50 ± 16.10 (24-86) years. Sixty-six patients were male (67.3%) and thirty-six were diabetic (36.7%). The GFR was estimated at 6.05 ± 2.36 and 5.83 ± 2.19 cc/min by MDRD and cystatin C based formulas, respectively. There was a significant correlation between the two formulas (r = 0.51; P < 0.001). Serum cystatin C level was 9.74 ± 2.47 mg/L which showed significant reverse correlation with both MDRD (r = -0.46; P < 0.001) and cystatin C based formula (r = -0.87; P < 0.001). Mean serum Creatinine before dialysis and eq Kt/V (eq) were 10.02 ± 3.16 mg/dL and 1.19 ± 0.40, respectively. Neither creatinine nor cystatin C showed correlation with Kt/V, as the marker of dialysis adequacy. The negative correlation between serum cystatin C level and age was shown (r = -0.32; P = 0.007) (Table 1).

**Table 1.** Baseline Characteristics of Patients (n = 98)

Variable	mean ± Std.D (Range)
**Age, y**	55.50 ± 16.10
**Dialysis Duration, mo**	58.93 ± 58.65 (4-228)
**Albumin, g/dL**	3.9 ± 0.4 (2.7-5.1)
**Total cholesterol, mg/dL**	149.7 ± 42.7 (16-292)
**Triglyceride, mg/dL**	151.3 ± 85.4 (5-573)

## 5. Discussion

In this study, accuracy of two equations derived from prior studies was examined. According to our basic knowledge, the range of GFR in functionally anephric patients is less than 10 mL/min. This study showed that cystatin C based equation can be used similar to MDRD formula for estimating GFR in dialysis patients.

Hoek et al. investigated in cohorts of both hemodialysis and peritoneal dialysis patients whether or not cystatin C based equation was able to estimate GFR more accurately than MDRD formula. This study showed that cystatin C measured before the dialysis was significantly correlated with mean creatinine (r = 0.505; P < 0.001) (13). Consistent with our findings, Guido Filler introduced cystatin C as a useful marker for monitoring children under dialysis (14). Similarly, correlation between cystatin C and Kt/V (sp) in patients on conventional or nocturnal hemodialysis was not found in other studies. However, they could show its correlation with standard weakly Kt/V (12, 14, 15), which may support the promising role of serum cystatin C level as a rough estimator of dialysis adequacy and residual renal function.

Our study had some limitations: the first was absence of measured GFR by a gold standard such as 99mTc-DTPA scintigraphy to assess relationship of cystatin C or creatinine based formulas. Nevertheless, it was shown that chronic kidney disease epidemiology collaboration (CKD-EPI) creatinine based formula and Hoek cystatin C based formula had the best correlation with 99mTc-DTPA in GFR of less than 15 cc/min (16). Secondly, we did not measure cystatin C after dialysis to investigate whether or not its level was affected by dialysis removal.

Serum cystatin C is related to creatinine, significantly; therefore, it can be considered as an indicator of renal function in patients with reduced GFR. According to significant correlation of GFR estimation by cystatin C and MDRD formulas, this study verifies its capability to be used interchangeably. However, serum cystatin C does not seem to be able to show dialysis adequacy.
